# Elderly Gravida with Bombay Blood Group and Placenta Previa Managed with Autologous Blood Transfusion

**DOI:** 10.1155/2020/8850500

**Published:** 2020-12-01

**Authors:** Pooja Paudyal, Geeta Gurung, Ashmita Adhikari, Suvana Maskey, Josie Baral

**Affiliations:** Tribhuvan University Teaching Hospital, Institute of Medicine, Kathmandu, Nepal

## Abstract

The Bombay blood group is a rare blood type with an incidence of around one in a million. There is no known reported case of an obstetric patient with the Bombay blood group from Nepal. People with this rare blood group can receive blood only from those with the same blood type. We report an elderly gravida with the Bombay blood group who had a pregnancy complicated by diabetes, placenta previa, and transverse lie (back up) following an in vitro fertilization. Placenta previa posed a greater risk of hemorrhage and hence the need for transfusion. The main challenge was arranging blood for transfusion, and as the Bombay blood group was unavailable, she was managed with autologous blood transfusion which was performed for the first time in a pregnant lady in our institute. She underwent Cesarean section for placenta previa with transverse lie, and both mother and baby were sent home in good health.

## 1. Introduction

The Bombay blood group also known as h/h or Oh is an extremely rare blood type, first reported in Bombay by Dr. Bhende et al. in 1952 [1]. It occurs in about 1/10,000 individuals in India and in about 1/1,000,000 in Europe [2]. It is characterized by the absence of A, B, and H antigens on red blood cells (RBCs) and presence of anti-A, anti-B, and anti-H in serum. As these individuals have antibodies in plasma reacting with all ABO phenotypes, they can receive blood only of the Bombay blood type [1, 2]. Apart from the unavailability of blood, the presence of placenta previa and transverse lie further complicated our case as maternal hemorrhagic morbidity is higher in women with previa (19% vs. 7%; aRR 2.6, 95% CI 1.9-3.5) and need for blood transfusion (12.9% vs. 3.1%; aRR 3.8, 95% CI 2.5-5.7) [3]. Owing to the rarity of the Bombay blood group, this case report is aimed at adding to the very scarce literature apart from discussing the challenges in this case.

## 2. Case Report

We report a case of a 41-year-old G3P2L1 lady of Indian descent, married to a Nepali with the rare Bombay blood group. A written informed consent has been signed by the patient allowing any case details to be published. Based on our hospital policy, Institutional Review Board (IRB) was not required as it was a case report.

Her first two children were born at home without any complications; however the second baby had died after a few days. Following this, she had a history of secondary subfertility with endometriosis for 10 years and had also undergone myomectomy in India 2 years back. She had then conceived following in vitro fertilization (IVF). She was hypothyroid, had developed gestational diabetes, and was under insulin therapy. At 35 weeks, she had an episode of per vaginal bleeding and was admitted to a hospital in Raxaul, India. Blood grouping revealed her to be O positive, but on crossmatching, her blood failed to match with any other blood type. Detailed evaluation with forward as well as reverse blood typing diagnosed her to have the Bombay blood group. In view of her high-risk pregnancy and nonavailability of the rare blood, she was referred to our centre. Her vitals were stable on arrival, and her hemoglobin was 11.4 g/dl. Ultrasonography revealed a fetus of 34 weeks in transverse lie with back up and complete placenta previa. Fortunately, it was not complicated by the placenta accreta spectrum as no myometrial invasion by the placenta was detected on Doppler study. Her blood type was reconfirmed in our blood bank and in the central blood bank of Red Cross Society. There was no availability of stored blood or any record of donor with this blood group in Kathmandu. This led us to plan for autologous blood transfusion with an informed consent from the patient and her husband. After building the patient's hematocrit with iron sucrose infusion, 350 ml of patient's blood was drawn and preserved on two occasions, 1 week apart, at 35^th^ and 36^th^ gestational weeks. Continuous maternal and electronic fetal monitoring was done during phlebotomy anticipating fetomaternal complications, and the periphlebotomy period was uneventful. Her hemoglobin following second phlebotomy was 10.5 g/dl.

She was planned for a Cesarean section (CS) at 37 weeks, but a day prior to that, she had per vaginal bleeding, so emergency CS was done. Baby was delivered via a J-shaped incision on the uterus as the lower uterine segment was poorly formed likely due to transverse lie. Hemostasis was secured with multiple hemostatic sutures at the placental bed and use of oxytocics. Blood loss during surgery was 700 ml. She was transfused with one unit of her stored blood, and posttransfusion hemoglobin was 9.5 g/dl. The 2670-gram male baby developed early-onset neonatal sepsis and was admitted in the Neonatal Intensive Care Unit (NICU) for a week. The baby improved with antibiotics and supportive care. The mother and baby were discharged in good health on the 12^th^ postoperative day.

## 3. Discussion

The Bombay blood group is a rare autosomal recessive phenotype within the ABO blood grouping system which occurs due to point mutation of the H gene that produces H antigen on RBCs. These people lack A, B, and H antigens in their blood and hence produce anti-A, anti-B, and anti-H antibodies [1, 2]. The absence of A and B antigens mimic the O blood group but the presence of anti-H antibody causes crossreaction with all blood types including the O group which carries H antigen so identification of the Bombay phenotype often occurs during crossmatching [1, 2]. Consequently, these patients can only receive either autologous transfusion or blood from another person with the Bombay blood group as mismatched transfusion can result in life-threatening events [4].

The Bombay phenotype has the highest prevalence in India, and many of the case reports in literature are from India. Saradabai and Sujatha reported a case similar to ours of a 28-year-old primigravida at 28 weeks with antepartum hemorrhage due to major placenta previa. She was transfused one unit of blood donated by her maternal uncle with the Bombay blood group and two more units from the blood bank [5]. Deo et al. report a case of a primigravida of Sri Lankan origin with the Bombay blood group diagnosed during antenatal evaluation [6].

There is no reported case in literature of a Bombay blood group from Nepal, and its prevalence in Nepalese population is unknown. Our primary concern in this case was the arrangement of blood for transfusion as she had placenta previa which put her at high risk for hemorrhage. We also anticipated difficulties at surgery and increased blood loss due to adhesions as she had history of myomectomy and endometriosis. Autologous blood transfusion was planned due to unavailability of homologous blood of the same group. Autologous blood transfusion entails storage of one's own blood for subsequent infusion if needed. It is widely performed in scheduled operative procedures to avoid or reduce risks of homologous blood transfusion [7]. Priye et al. described autologous blood transfusion in a 45-year-old patient with the Bombay blood group undergoing mitral valve replacement. Three units of autologous blood was collected over three weeks and transfused in the postoperative period [8].

Autologous blood donation during pregnancy is not as commonly done as in nonpregnant since it can in itself lead to maternal and fetal complications like exaggeration of anemia in the mother, vasovagal symptoms, and fetal heart rate abnormalities. Several studies have shown autologous blood storage during pregnancy to be relatively safe for both mother and fetus and to be feasible transfusion practice [9]. Others however proposed the need for reappraisal in obstetric patients [10]. Yamada et al. performed autologous blood donation and storage in 32 patients with placenta previa with almost no complications. They recommend starting blood collection at 32-week gestation with phlebotomy of 400 ml per week so that a total of 1200–1500 ml of blood can be collected and stored [11]. We were able to collect 700 ml of blood through two donations as patient presented late at 35 weeks.

Apart from iron supplementation and preoperative autologous donation, acute normovolaemic haemodilution and the use of cell salvage system are other alternatives to allogenic blood transfusion [12]. But due to unavailability of cell salvage system, we opted for autologous donation.

## 4. Conclusion

This was a challenging case as we did not have any previous experience of the Bombay blood group and autologous blood transfusion in pregnant women. However, we successfully managed her with autologous blood transfusion with good maternal and fetal outcome.
